# The ‘Paths to everyday life’ (PEER) trial – a qualitative study of mechanisms of change from the perspectives of individuals with mental health difficulties participating in peer support groups led by volunteer peers

**DOI:** 10.1186/s12888-024-05992-w

**Published:** 2024-08-13

**Authors:** Cecilie Høgh Egmose, Chalotte Heinsvig Poulsen, Siv-Therese Bogevik Bjørkedal, Lene Falgaard Eplov

**Affiliations:** Copenhagen Research Unit for Recovery, Mental Health Centre Amager, Hans Bogbinders Allé 3, 3. Floor, Copenhagen S, 2300 Denmark

**Keywords:** Peer support, Volunteer peers, Civil society, Personal recovery, Mental health, Mental illness

## Abstract

**Background:**

Worldwide, peers support has been shown to play a crucial role in supporting people with mental illness in their personal recovery process and return to everyday life. Qualitiative studies underpinning the mechanisms of change in peer support has been reviewed. However, the findings are primeraly based on the perspectives of peer support workers employed in mental health services. Thus, qualitiative studies elucidating the mechanisms of change from the recipient perspective in mental health service independent civil society settings are higly needed to further contribute to the evidence of peer support. The ‘Paths to every day life’ (PEER) is evaluated in a randomized trial and is substantiated by qualitative studies investigating the experiences of PEER from the perspectives of the recipients and the facilitators of peer support. The purpose of this qualitative study underpinned by critical realism was to substantiate the PEER intervention program theory by gaining deeper insight into the change mechanisms and elaborate how, when, and under what circumstances the peer support groups potentially had or did not have an impact on personal recovery from the perspectives of the recipients of peer support.

**Methods:**

Eleven individuals were interviewed at the end of the ten-week group course. The semi-structured realist-inspired interviews were audio recorded and transcribed verbatim. The analysis was guided by reflective thematic analysis and through an abductive framework based on the program theory. Data were coded and analysed in Nvivo software.

**Results:**

Four overarching themes were identified that informed and nuanced the program theory: 1) Connectedness as a prerequisite for engagement; 2) A sense of hope by working out new paths to recovery; 3) Seeing new sides of oneself; and 4) Sprout for change.

**Conclusions:**

This study substantiates the program theory and the quantitative results of the PEER trial by elaborating on mechanisms that were felt to be essential for the personal recovery process from the perspectives of the recipients of the group-based peer support. In addition, the study points out that the opportunities to act in everyday life depended on individual context and where the group participants were on their recovery journey.

**Trial registration:**

ClinicalTrials.gov identifier: NCT04639167.

**Supplementary Information:**

The online version contains supplementary material available at 10.1186/s12888-024-05992-w.

## Introduction

Mental health is the foundation for individual well-being and a fulfilled productive life. However, worldwide, mental health difficulties and mental illnesses are highly prevalent and have severe impacts on people’s everyday lives, leading to mental distress, occupational- and social disabilities, and experiences of being stuck in life [1]. In Denmark, the majority (82%) of individuals will either receive a diagnosis of mental illness or will be prescribed psychotropic medication during their lifetime [2]. Mental illness has major human and socio-economic consequences [3]. Consequently, increasing efforts to promote mental health and support people with mental illness in their recovery and return to everyday life is urgently needed.

Peer support defined as “giving and receiving help founded on key principles or respect, shared responsibility and mutual agreement of what is helpful” [4, 5] delivered in various forms by individuals with lived experiences of mental illness is regarded as a central element in recovery-oriented practices [6–11]. In this paradigm, the concept of personal recovery is defined as “a way of living satisfying, hopeful, and reciprocal lives, together with others even though we may still experience distress…”[12]—the concept differs from clinical recovery, which has traditionally focused on the reduction of symptoms and increased levels of functioning. Despite a growing interest and implementation of the various types of peer support, the evidence about its effectiveness is mixed. Results from meta-analyses have shown that individual and group-based peer support added to mental health services modestly improve personal recovery and decrease some psychiatric symptoms e.g., anxiety among individuals in treatment for severe mental illness (SMI) [13–16]. Nevertheless, questions have been raised whether the core values ​​of peer support delivered within mental health services contexts can be maintained [14, 17].

In the critical realist approach, context is considered as social rules, values, sets of interrelationships’ that operate within times and spaces that either constrain or support the activation of programme mechanisms [18, 19]. Thus, alternatively, peer support delivered outside the mental health systems has been proposed to have better contexual conditions for maintaining the core values of equal relationships, equal power balance and a focus on personal recovery rather than clinical recovery [17]. A few randomized controlled trials (RCTs) have shown that peer-designed group-based interventions delivered in community settings modestly improve self-advocacy among individuals with SMI [20–23]. However, as proposed by the British Medical Research Council (MRC) for evaluating complex interventions the RCT design does not allow for a deeper understanding of how outcomes can vary across contexts and how change mechanisms might lead to outcomes. Therefore, process evaluations with qualitative studies investigating mechanisms of change hypothesised in a program theory are highly recommended to nuance the effectiveness of the various types of peer support [24, 25]. Nevertheless, theoretical models [26] and qualitative reviews [27, 28] proposing underlying change mechanisms in peer support include more accounts from the providers than from the recipients of peer support. Furthermore, most qualitative studies have been conducted in the mental health services, while peer support received in civil society settings might be different but is less investigated.

To add to the evidence base, and to contribute to the scientific knowledge about peer support delivered in a mental health system independent, community-based context, a multi-center RCT with high methodological quality was conducted between 2019–2023 [29]. PEER was cocreated in a close collaboration between the Peer Partnership Association (non-governmental organisation (NGO)) and researchers with lived experiences of mental illness. Thus, it is a co-produced evidence-, practice-, trauma- and lived experience informed 10-week manualized group course based on the needs of adults with mental health difficulties – defined in this project, as persons who is affected by mental health dissatisfaction to a degree that limits their unfolding of life with or without an assigned psychiatric diagnosis. Participants were recruited from Danish municipality social services and through self-referral. Inspired by the MIND organization in UK [30], two volunteer peers with lived experiences of mental illness were recruited by the NGO to co-facilitate the groups in collaboration. The choice of a voluntary co-peer model, was to ensure power equality in the group community and promote a clearly defined and supported peer role, as well as to promote a safe space for the participants who were ensured system anonymity. For an in-depth understanding of if and how the PEER intervention potentially promoted changes towards recovery and how the intervention interacted with contextual factors in a civil society setting, a qualitative study underpinned by critical realism [31, 32] was launched, in conjunction to the RCT.

In this qualitative study, we aim to investigate the mechanisms of change of the PEER intervention and elaborate how, when, and under what circumstances the group dynamics, the elements, and exercises in the intervention potentially have or did not have an impact on recovery and well-being from the perspectives of the group participants. Secondly, we aim to identify connections between intervention elements, context, group participants, mechanisms and outcomes in line with our findings to revisit and revise the program theory.

## Material and methods

This qualitative study is designed to evaluate the program theory and substantiate the quantitative results of the PEER trial (manuscript in preparation) in a process evaluation framework [24, 25] focusing on the participants’ experiences of the group-based peer support delivered in PEER [29]. The PEER trial has been registered on ClinicalTrials.gov (NCT04639167, registered November 19, 2020) and ethically approved by the regional ethics committees of the Capital Region of Copenhagen (H-20027612). This study is following the consolidated criteria for reporting qualitative research (COREQ) [33], The participants signed informed consent before the interview.

### Theoretical framework

The qualitative study is underpinned by critical realism as the philosophy of science [31, 32]. In the critical realist approach, causal mechanisms are social structures that can be understood through and exist within phenomena at the empirical and actual level e.g., human actions, opinions, and ideas that are generated by these mechanisms. Conditions in the open social world can prevent or facilitate the actualization of a social structure’s causal power [34]. Therefore, the awareness of context i.e., social rules, use of language, values, interrelationships etc. is considered important in the interpretation of mechanisms [18]. Inspired by the realist interview approach and using the concept of teaching and learning, the interviewers explicitly stated which experiences they wanted to learn about and asked the participants if they wanted to elaborate on this in order to mutually learn from each other in an iterative process [35]. According to this approach, the program theory i.e., hypotheses about how the PEER intervention elements and activities potentially contributed to change were drawn and explained from a printed initial program theory (additional file 1), while maintaining an exploratory openness towards the participants’ experiences and point of view throughout the interview in order to learn from the participants. The data analysis was based on reflective thematic analysis [36], and informed by a change model of peer support [26] suggesting [36]theories on social learning and social comparison [37–39]. Furthermore, we searched for generalizable patterns between intervention elements, contextual conditions, actors e.g., the group participants, underlying mechanisms and outcomes using retroduction from critical realism [40]. 

### The Paths to everyday life (PEER) intervention

PEER is a person-centered civil society-based and manualised intervention designed based on the needs of individuals with mental health difficulties described in detail elsewhere [29]. In addition to ten group-based sessions led by two voluntary peers trained in the manual, PEER also offers individual companionship for up to six months to local communities and activities outside the group setting. The content of the group sessions is based on themes developed from the CHIME (Connectedness; Hope, Identity; Meaning; Empowerment) framework [42], life storytelling [43], and the mindset of acceptance and commitment therapy [44]. Before the course there is an obligatory introduction meeting to inform participants about the group. PEER aims to support people with mental health difficulties by forming a constructive community with possibilities to exchange lived experiences, experience mutuality, and develop social network. The anticipated change mechanisms in PEER are shown in the initial program theory (Additional file 1) and cover reciprocal sharing, acting in own life, as well as providing participants with opportunities and abilities for bridging to and engaging in the wider community, which downstream may lead to increased quality of life, wellbeing, and personal recovery.

### Recruitment

Participants were recruited between August 2021 and February 2022 through information flyers handed out by either peer group facilitators or local coordinators at the municipality study sites. The recruitment strategy aimed for variation concerning study-site, sex, education, and age, completers and non-completers. Hence, the sampling method was purposive [45]. The sample size was determined from the concept of information power that indicates the more information the sample holds, relevant for the overall study aim, the lower number of participants is needed [46]. Thus, the initial assessment of the sample size was 9–12 participants. As recommended, an early assessment of information power was made after the first five interviews, which guided the recruitment strategy and revision of the interview guide. The final assessment of information power indicated that the data from the interviews with eleven participants was sufficient to address the aims of the study. Two participants who initially agreed to participate were not interviewed due to cancelation from their side. All participants signed informed consent before the interview.

### Data collection

Semi-structured realistic inspired interviews were conducted at the end of the 10-week PEER group course. Lived experience researchers (CHE; CHP) used their professionalism in the research field of mental health promotion, as well as their own lived experiences of mental illness and recovery actively, e.g., through the understanding of the field, and explicit during the research process from stipulating research questions within a program evaluation context, developing the interview guide, in the interview situation e.g., disclosure about having experiences with mental illness to analysis and interpretation of results. After each interview field notes were made. The interviews took place in locations according to the participants’ preference and varied in length (40–60 min.). The interviewers initiated the interviews by disclosing their own lived experiences of mental illness, as well as their professional point of view. After four interviews, the interview guide was reviewed. A few changes were made to better capture the information desired concerning the research aim, and to gain information power [46]. Another review of the guide was performed after a further four interviews. Topics in the interview guide were founded on the original PEER program theory to elicit responses to answer the research aims, and to gather information about participants and their context (Additional file 2). Interviews were audio recorded and transcribed verbatim using the Nvivo version 1.7 (QSR International) transcription program. Transcripts were not returned to participants for comment correction due to time constraints.

### Method for analysis

**Fig. 1:**
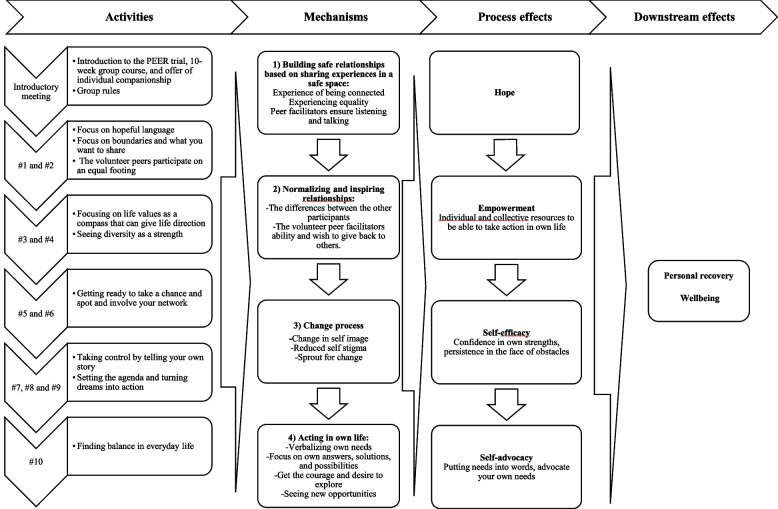
Revised program theory of the ‘Paths to Everyday life’ (PEER) intervention

The analysis was guided by the six phases of thematic analysis [36, 47, 48]. In line with the critical realist approach, the initial code tree was generated from the interview guide and program theory, as well as codes regarding context. The coding process was flexible abductive and based on the initial program theory that was inspired by the change model of peer support underpinned by e.g., social learning theory and social comparison theory [26]. E.g. deductive codes such as ‘education/work-’ and ‘illness-history’, as well as ‘group relationships’, ‘role modelling’, ‘acting in own life’ and ‘social attachment’. However, with a simultaneous openness to capturing inductive codes such as ‘fit into the group’, ‘connecting’ and ‘others are worse of’ [36, 47]. The initial coding was conducted by the lived experience researchers (CHE; CHP), independently, and subsequently compared, and discussed. The remaining coding was conducted by CHE. The codes were used to search for demi-regularities [34], and collected into themes in an initial thematic map. The demi-regularities and raw themes were presented with data extracts and discussed with co-authors (SBB; LFE), and at a workshop involving representatives from the NGO that provided PEER i.e., the lived experience lead project manager, one volunteer peer and three local peer coordinators. Notes were taken and used in the process of finalizing the themes, subthemes, and mechanisms. In the final step, mechanisms were identified and connected to prior theory to deepen our understanding of the PEER intervention. In this phase, ICAMO configurations were searched for and identified. The results are presented as overarching themes. In the discussion, findings are reviewed using the concepts of ICAMO configurations, and integrated into the revised program theory of PEER (Fig. 1).

## Results

Eleven participants from four study sites were interviewed between December 2021 to April 2022. Nine completed the group course and one of them also received individual peer companionship. Two participants left the group after 1–2 group sessions. Participant characteristics are shown in Table 1. The majority of the participants were aged 30–50 years, had received a psychiatric diagnosis at some point in their life, lived alone, had 2–4 years of higher education, and few were working.

**Table 1:** Characteristics of the participants obtained from the interviews

Participant	Sex (age)	Partner	Children	Type of education	Occupational status
1	Female (45–50)	No	Yes	Vocational	Job seeking
2	Female (45–50)	No	No	-	Pension
3	Male (50–55)	No	Yes	Vocational	Working
4	Male (40–45)	No	No	Vocational	Social benefits
5	Female (25–30)	No	No	Bachelor	Job seeking
6	Male (35–40)	No	No	-	Job seeking
7	Female (25–30)	Yes	No	Bachelor	Working
8	Female (30–35)	Yes	No	-	Job seeking
9	Female (30–35)	Yes	No	Vocational	Pension
10	Female (60–65)	No	Yes	Bachelor	Pension
11	Female (45–50)	Yes	No	Vocational	Pension

Four overarching themes and some subthemes were identified, elucidating how, when and under what circumstances PEER impacted recovery and wellbeing, from the perspectives of the participants: 1) Connectedness as a prerequisite for engagement; 2) A sense of hope by working out new paths to recovery; 3) Seeing new sides of oneself; and 4) Sprout for change.

## Connectedness as a prerequisite for engagement

Most participants described how they felt a connectedness in the group. Conversely, those who discontinued the intervention, described how they did not feel connected to the other participants or the peer facilitators. This was a part of the reason why they chose to leave. Connectedness within groups mattered because loneliness and not feeling connected to others was something many participants struggled with.

*“Loneliness was actually for all of us, that about not feeling connected or feeling half empty or feeling that we were ripped over or missing someone to fill it. But it was awesome to articulate it yesterday, that it has been there all the way through, and it hasn’t been taboo. It has been able to fill as much as it should on the particular session where someone has said, Auch, I feel alone”* (Participant 1).

Not feeling connected to the group was described by one participant:

*“I did not experience it as safe. I feel. I feel miscast, simply. Yes, so it is also connected to why I have chosen to stop. And yes. So I’m not. I have not found it meaningful to be with those who feel that way. No. Simply because I don’t feel I fit in.”* (Participant 7)

Lack of connections to the other participants in the group was also attributed to differences among group participants concerning e.g., having a job or age. Participants who remained in the group did not perceive differences as a barrier to connectedness. Listening to others sharing experiences increased the feeling of a safe space. Also, acceptance and non-judgment were part of feeling connected and safe to share experiences in the group.

### Creating a safe space to connect with others

Feeling safe in the group was very important for engaging in PEER. Several aspects mattered for whether participants felt safe or unsafe e.g., the size of the group. A participant felt misplaced by the group facilitators and that made her feel uncomfortable, and this was part of her reasoning for choosing to leave the group.

*“That was the primary reason why I stopped. It was, as it were, the two’s [peer group facilitators] way of dealing with that, and not being able to create a safe space. Because when there are people, who are having a hard time, when there is just complete silence, and you can see that everyone.”* (Participant 1).

The peer facilitator’s role was highlighted as key in creating a safe space for sharing. E.g., For the initially agreed group rules such as everyone getting speaking time; showing each other spaciousness; speaking up if there’s something you don’t want to share, etc. also created a safe space at the group sessions. Participation in the group was anonymous and confidential meaning that no one reported anything back to anyone outside the group setting. A safe space was important for participants, to find the courage to share their experiences and to engage in the intervention.

### Consolidating connectedness by sharing experiences

Sharing experiences was brought up by all participants as a vital part of being in the group. Through sharing experiences participants felt safe and connected to others. It enhanced a feeling of being understood, and not being alone, and provided participants with opportunities to learn from others. One participant described:

*“The thing about meeting another person there, where you can look in the eyes, that person has tried to be there, and there is zero light. yes, and then that magical recognition happens, the plane of understanding, the level. Hey… You have felt the pain, we both have a pain in common.”* (Participant 1)

Most participants perceived the sharing of experiences in the group as positive. However, a few felt it was difficult and that sharing made them feel exposed. Some participants described how they as children had been bullied and maybe therefore had bad experiences in being vulnerable in front of others. Self-determination was an important aspect of sharing experiences. Participants described that it was accepted when they chose not to share. The active involvement of peer facilitators in sharing experiences created equality in the group which in turn could lead to more sharing and getting to know one another:

*“It was super nice. There was no one higher on the ladder than others as such. It was super nice. We got to know each other a little better that way.”* (Participant 5)

Peer group facilitators were also perceived as equals by the participants because they were peers and not professionals. The fact that peer facilitators were voluntary, thus unpaid, made them more equal in the group.

### Sense of hope by working out new paths for recovery

Hope was described by participants in different ways. For some, the feeling of not being alone gave hope. Others felt inspired by the peer group facilitators. Seeing them do volunteer work, introduced new possibilities for the participants. Some described that they during PEER had gained new beliefs that everything was going to be all right.

*“And in principle, I don’t know anything as such about how and how my entire future will of course shape itself. But [..] I see it as a new, new possible life, I see that, going forward here. And I’m hoping for that, that is, to get a good second half instead of the first half, where I’ve just been struggling and fighting my way through for as long as I can remember. So maybe a little less fight and a little more life.”* (Participant 2)

A part of this was paying attention to whether there were any dreams or wishes regarding the future. Hope was nurtured when learning about the group facilitators lived experiences e.g., that it was possible to get a flexible job despite mental health difficulties. One participant mentioned a group exercise, that focused on dreams:

*“There were many simple things, for example, what are my dreams, my dream is to travel to Rome and Jerusalem. In other words, if I hadn’t been put that question directly in the face, then I probably wouldn’t have come, then I probably wouldn’t have expressed it…”* (Participant 4)

Namely, being accepted into the group strengthened the participant’s hopes that this will also be possible in the future. A participant who struggled with drug addiction described how her dreams of being clean gave her hope for the future.

### Seeing new sides of oneself

Several of the participants had a narrative about themselves as being vulnerable and unemployable, both concerning paid and voluntary work. Through life storytelling and by comparing themselves to others in the groups, seeing that some were worse off, participants revised their self-perceptions and discovered new aspects of themselves during PEER (Participants 2, 4 and 5). Several participants talked about their diagnosis and how it impacted their self-image. For one participant receiving a diagnosis of autism was perceived as beneficial, since it had led to a more positive and accepting self-understanding. For others, the diagnoses were a part of the narrative about illness and limitations. Participants reported that the life storytelling exercise gave a deeper self-understanding (Participants 3, 4, and 5), as well as a path to increase their knowledge about others. Telling one’s story was a mechanism for changing self-image and gaining acceptance and recognition (Participants 4, 5, 8, and 10). Furthermore, it was described as a path to bridge the past with a focus on a way forward. One participant stated that life storytelling had been painful because it became a story about illness and limitations. However, she also realized that the story could be told differently with a different focus:

*Well, we talked about that afterward, so when we had to pick up. I would like to rewrite my story a bit. Yes, so I had more focus on some of the positives and some of the good. Because there have been good things [..] So, at least that was the exercise I could take home with me. What should my life story look like, from now on, so that I don’t just place, paint myself as if I’m just sick.”* (Participant 5)

PEER participants learned that shifting the emphasis of one’s life story could result in a shift in one’s self-image toward better self-appreciation, a sense of being worthy, and an understanding of one’s resources.

*“No, I have found my inner strength again [..] That thing of constantly telling myself that I’m not worth anything and that I’m not good enough. Why should people tell the truth when they say they like me?”* (Participant 1)

Changing perspectives during PEER was enabled by elements in the intervention but also changes in the participant’s life circumstances. For instance, one participant retired, and another one was in the process of addiction rehabilitation. One participant described how trying something new in the safe and accepting atmosphere of the group, made her feel less helpless and more capable:

*“But I can, there are just some days when I feel a little bad mentally, and I know that I just have to prove to myself that it’s not every day that I necessarily feel so bad mentally that I can’t do it myself. Then I needed, I had to have the courage to show it. Me, I’m not as helpless as I always think I am.”* (Participant 8)

### Others are worse off

Several participants compared themselves to the other group members, which gave them a perspective that some of the other participants were in a worse situation than themselves. The comparison gave them a perspective on their situation and resources:

*“No, then it’s because it gave me such a.. are. okay. Some have it significantly more difficult. And it was also a bit depressing, you could say. To meet some who are completely stuck in life. But it also gives you, such a [..] You might be ok. strong, nonetheless. It was kind of both ways in that.”* (Participant 7)

Through comparison with other group members, the participants´ self-image and identity were clarified and put into perspective. Two of the participants who made this statement were employed and the other participants in their groups were not. In these situations, the comparison led to an increased positive view of oneself and one’s resources and abilities in their individual context.

### Sprout for change

Participants frequently cited a wish for change as the reason they joined PEER; they were either in a situation where they had a specific desire for something to change or they wanted to see whether the PEER participation could enable a change process:

*“That’s also why I chose it because to feel different, I felt a bit like, beside myself at the time, when, before I entered the group.”* (Participant 11)

During PEER, participants found inspiration for making changes in their lives. The participants had different perspectives on what change, if any, they had gained from attending. Most participants felt they had gained something from participating in the group but without being able to be more specific. One described how participation had given him a space where it was possible to talk about difficult subjects:

*“But I find it difficult to put into words what I can take with me, what I can use. I don’t know what to answer to that. I’m pretty sure I have brought something, yes, but just putting it into words, I don’t know.”* (Participant 3)

Some described the unspecific change as a ‘sprout for change’:

*“But I still feel that, as I said, that there is no doubt that it has put some sprouts [..] under the skin, and what can I use this for?”* (Participant 2).

According to the participants, the timeframe of the group was important to consider when talking about change. The short time aspect was highlighted as a factor for not being able to point to a specific change, as change was viewed as taking more time.

*“Because ten weeks it helps to create some sprouts, yes, but those sprouts must also be watered [..] because I think ten weeks is fine enough to start something. And of course, that, that’s the goal, that something is started with personal development about recovery and quality of life, but what about in a month?”* (Participant 4)

The group sessions with their themes and exercises can be viewed as mechanisms for starting a change process, which can be seen as steps towards personal recovery. However, changes for the participants seemed to take more time. Nevertheless, the descriptions of gains indicate that even a relatively short intervention might have an impact on the participant’s life. Some participants described a change in how they viewed their ability to do voluntary or paid work. Several participants wished to help others or to make a difference for others and viewed this as meaningful. The change was perceived as a realization that paid or voluntary work was possible if the right niche and environment were found:

*“I’ve always wanted to help people and have had different things I wanted to do concerning that. Which then couldn’t be done because I got sick. Saw some of the things and then I’ve heard a little more about them and just thought [..] maybe it’s not such a good idea after all.”* (Participant 5)

*“Yes, is it something that has given you more courage to do it now?* (Interviewer)

*“Yes, I think so. That. It’s just finding your niche, something where you can volunteer and make a difference.”* (Participant 5)

Through engagement in PEER, a sprout was planted to believe that voluntary work was a possibility. Another participant described how she had found out where she wanted to work and that her case worker supported her in realizing it. Additionally, two participants described how the group session changed their consumption of alcohol and drugs because they wished to participate and would not be able to do so if intoxicated. So even though the participants in many instances could not specify changes when asked, the sprout for changes was seen through descriptions of their experiences in the group.

Specific changes were described concerning the exercises in the group sessions. The exercises were about making a change and trying something new, acting, and defining hopes and dreams. The process of change was described as difficult. Some described how they lacked ideas about how to make a change (Participants 3 and 5). Others described it as a good learning experience. For one participant, the group exercise about making changes made a big difference, enabling her to take more control over her own life and financial situation:

*“Took some chances and canceled my apartment, just like asked him to move. Have done something about my finances, and take far fewer drugs than I did here [indicate a point in time in the group course program]. Yes, it has also had an impact on [..] Yes, it is simple because I have been inside and taking this thesis that I keep talking about, the control of my own life back in a way where that it has become even more important for me to reach something where I get more balanced.”* (Participant 1)

This was important to the participant’s life because of unemployment and low income due to social benefits, and she had almost spent all her savings on living in a too-expensive apartment. Another participant described how the exercise made her try going out to a grocery shop on her own, which she did not do otherwise. Succeeding in this gave her the courage to take new chances and made her realize that she had resources. Making changes worked as a mechanism for trying something new, as well as the experience of success with something new, which in turn led to courage to try more new things, which led to a change in the view of own resources. Thereby, the PEER intervention did lead to changes for most of the participants. For some, the changes were obvious as they were directly linked to the intervention exercises. For most the mechanisms were an interaction with the whole intervention, the other mechanisms and connected to individual context, the context of the municipality, and the societal context of Denmark e.g., occupational and social laws, culture, etc.

## Discussion

### Summary of key findings

This study aimed for a deeper understanding of how PEER was experienced by the participants i.e., how, when, and under which contextual circumstances the group dynamics, the elements, and exercises in the intervention potentially had an impact on recovery and well-being. Results showed that finding safety and courage to share experiences in the group were fundamental mechanisms for relationship-building connectedness and group cohesion. When these mechanisms were not given time to be activated, it contributed to participants leaving the group. These mechanisms were enabled by the voluntary peers, through their sharing and facilitation of the group in civil-society settings in-dependent of the mental health service- and social systems, where most of the participants had previous experiences. For participants, the group context withs its social rules, values and interrelationships led to feelings of not being alone and being accepted as a person, which led to a belief in a better future that they had not experienced in other settings. An unexpected finding was that comparing oneself to others, both those who are well, but also those who are worse off, might change one’s self-image and life narrative in a positive view possibly a mechanism contributing to reduce self-stigma. Change processes during PEER were both results of engaging in the group, as well as specific intervention elements and exercises. The findings illustrate how important it is to consider the mechanisms that underpin the intervention, as well as the context in-dependent of systems and the actors e.g., the equal relations between the group members and the voluntary peers since it played a vital role in outcomes generated – intended or unintended.

The findings of the study supported previous research on mechanisms of change in peer support highlightning the importance of trustful relationship building in promoting the personal recovery process [26, 28, 42]. However, as previously mentioned, the contextual conditions for peer support provided in civil society by voluntary peers trained in an NGO are likely to have an impact on the maintenance of the core values such as equal power relationships, reciprocal support, and a ‘whole life’ rather than a ‘illness focused’ approach [14, 17]. Thus, our findings added that the peers were experienced as equals, because they were unpaid volunteers who participated and shared experiences and feelings equally in the groups, which provided shared experiences of hope, normalization and inspiration. These mechanisms may contribute to nuance the ‘role modelling recovery’ mechanism suggested in the literature [26]. Furthermore, the mechanisms are supported by another qualitative study conducted in a NGO setting, where the participants viewed individual peer support as especially valuable because of the opportunity of a non-treatment based normalizing mutual relationship that inspires hope [49]. Moreover, our findings are in line with a qualitative study of independent self help groups stressing how important it is to create contexts, which is experienced as a ‘safe space’ promoting trustful non-judgemental sharing, belonging and commonality [50]. These findings are an example of how contexts and mechanisms are enmeshed and operate in relation to each other [18]. Our findings did not confirm proposed mechanisms of ‘bridge building’ to the wider community (additional file 1) [26]. This is despite the attempt to facilitate bridging and engaging through the offer of individual companionship with a volunteer peer for up to six months to local communities and activities outside the group setting. Nevertheless, the offer was challenged by COVID-19 restrictions at the time of the PEER trial and only accepted by one of the interviewed participants possibly explaining why these potential mechanisms were not unfolded in this study.

### Revision of program theory and implications for research

Secondly, the purpose of this study was to propose realist-informed ICAMO configurations in line with our findings to revisit and revise our program theory. Our findings, although only based on interviews with eleven partipants, indicated four overarching ICAMO configurations, which informed the revised program theory (Fig. 1), and were inspired by examples and theory regarding realist evaluation [19, 41, 51]. The first ICAMO configuration relates to the building of safe relationships based on sharing lived experiences and how this is a condition for all the following ICAMO configurations to be activated. All participants described how sharing lived experiences was valuable. It was made clear that feeling safe was key to being able to share and this was created through the intervention elements and context such as the group rules, secured anonymity, the manualized form, and the core values of the intervention. When safety was established, it led to other configurations and experiences of connectedness, hope, a different view on their resources, and possibly increased the ability to speak up for themselves. The honest sharing of lived experiences can work as a tool for the concept of modeling from the social learning theory by A. Bandura [38]. In this perspective, the participants and the peer group facilitators can illustrate and inspire through examples of personal recovery or paths to everyday life what works for them in a live demonstration and those modeling influences can lead to behavioral change [26]. Still, the focus in PEER on the participants’ solutions and answers was important to ensure equality in the group as also highlighted by [28]. Moreover, according to the theory on social comparison by Festinger [37] one explanation for the lack of connectedness is the tendency to focus on differences rather than similarities with the others in the group. Through the lack of comparability to the other participants the group lost its value for the two participants who chose to leave the group.

The second ICAMO related to the experience of normalizing and inspiring relationships (Fig. 1), which was closely related to honest sharing, the differences between group members and peer facilitators, as well as the changed view on themselves. This finding had a changing influence related to the third ICAMO configuration developing a more positive self-image and reducing the self-stigma that emerged in most of the participants’ life-story narratives. This internalization of negative views about mental illness as being vulnerable and how it is connected to not being in the workforce can be seen in the light of the theory of N. Rose about thoughts on disorders without borders [52, 53]. N. Rose argues that it increasingly has become a way to govern from a distance that individuals internalize society’s ideas about the healthy and productive citizen [52]. Thus, the interviewed participants may internalize the perception of society that individuals with a diagnosis of mental illness are vulnerable individuals living with a disorder that needs to be treated [53]. However, the mirroring in other people had a changing influence on this internalized perception, which had the potential to empower the participants to make their own free choices.

The fourth ICAMO configuration relates to acting in own life, which was closely related to mechanisms of the change processes and the group exercises that for some participants led to a changed view on own strengths and resources. The aspect of change can be viewed through social learning theory as described by A. Bandura [38], where identifying an act of own choice, preparing for the act in the group, and getting non-judgmental feedback before and after the action can give courage and desire to try again. Also, Vygotsky’s theory on zone proximal development is relevant to understanding that learning awakens a variety of internal development processes that can operate when interacting with the environment and collaborating with peers [54]. All four IACMO configurations can be seen in the paradigm of personal recovery and the CHIME framework, which underpinned the language and the core values of the PEER intervention. However, a prior synthesis of qualitative literature has shown that not all experiences of personal recovery fit into the CHIME model [55]. Stuart et al. point out that it is important to broaden the perspective and incorporate contextual difficulties including internal and interpersonal struggles, financial problems, housing problems, negative perceptions and worries as part of the recovery process. This might explain that individual context and difficulties in life circumstances seemed to have an inhibitory effect on the change process for some of the interviewed participants. Additionally, the ability and consciousness to identify life values and thereby valuable actions, as well as the time point for the interview at the end of the ten week group course was identified as having an important impact on the change process – explaining the statements about sprouts for change.

The findings, as well as the suggested ICAMO configuration, led us to revisit and consider our initial program theory, which was based on perspectives of personal recovery within the project group, as well as knowledge about change mechanisms in peer support [26, 28]. Several aspects of our initial program theory, especially regarding the bridging and engaging mechanism did not seem to be activated as anticipated, as well as the outcomes of functioning, social network, and quality of life – thereby resulting in a refined program theory in the context of the municipality civil society-based PEER intervention (Fig. 1).

### Strengths and limitations

The strengths of this study relate to a consistent methodology according to critical realism, as well as the process evaluation framework [24, 25]. The lived experiences of the researchers (CHE; CHP) were a strength in connecting with the participants in the interview situation, as well as in the analysis process and interpretation of findings. The discussions with co-authors (LFE; SBB), as well as the involvement of persons employed in the NGO to carry out the operational part of the project were a strength as it brought further perspectives to the data analysis. The data collection across study sites, as well as the theorizing of the interview [19, 56] allowed for a deeper understanding of the interplay between intervention elements, group participants, contexts, mechanisms, and outcomes. Interviews with participants who chose to leave the intervention gave valuable insights and helped reduce the bias of participants who were positive towards PEER. Nevertheless, the study had some limitations e.g., it is important to mention that the suggested ICAMO configurations were based on interviews with eleven participants, why they should be interpreted with caution. Furthermore, the influence of context were not adequately unfolded in this study. To address this purpose, it would have been advantageous to add questions about the experience of context seen in relation to other contexts in the interview guide, e.g. previous experiences of other interventions, mental health services and social systems. Moreover, the study sites of the PEER intervention increased from three to five during the RCT trial phase. However, only participants from four sites were included in this study. Participant sampling was not as wide as intended since no participants under the age of 28 signed up for the interview even after a more targeted recruitment. One can also speculate whether the recruitment strategy favored those who still chose to be in the group and whether the peer group facilitators had a gatekeeper function. We addressed this limitation by introducing that the local coordinator could invite non-completers. Repeated interviews, as well as presenting findings for the participants were not conducted due to time and financial restraints. However, it could have given valuable insights into individual changes over time and participant views on data interpretation. Lastly, only one of the interviewed participants received individual companionship, which limited this study from exploring mechanisms of this part of the intervention, which could have impacted the participant’s opportunity to take new steps into e.g., other inclusive communities and occupational activities.

## Conclusion

This qualitative study examined how the PEER intervention was experienced from the perspectives of the group participants and proposed a revised program theorymodel describing change mechanisms and potential outcomes in the context of PEER. Findings showed that the participants in general found the PEER intervention beneficial even though not all participants were able to specify how. The findings also indicated that the PEER intervention with its manualized and thematic content was not for all. In general, this study contributes with valuable insights into how peer support is received in a co-peer facilitated manualized group format with a focus on being in the personal process of recovering together with others in similar life circumstances. PEER has the potential to facilitate connectedness, hope, change in self-image and initiate individual change processes through the major mechanisms of sharing experiences in a context experienced as a safe space, as well as willingness and courage to take action to try out new possibilities in life. Furthermore, it highlights some of the active mechanisms and contextual conditions, that are important in terms of creating and not inhibiting this change.

### Supplementary Information

Supplementary Material 1

Supplementary Material 2

## Data Availability

The data that support the findings of this study are available from the corresponding author but restrictions apply to the availability of these data, which were used under license for the current study, and so are not publicly available. Data are however available from the authors upon reasonable request and in accordance with Danish legislation.

## Abbreviations

CHIME: Connectedness; Hope; Identity; Meaning; Empowerment
COREQ: Consolidated criteria for Reporting Qualitative research
ICAMO: Intervention; Context; Actor; Mechanism; Outcome
MRC: Medical Research Council
NGO: Non-Governmental Organization
PEER: Paths to Everyday Life
RCT: Randomized Controlled trial
SMI: Severe Mental Illness
