# Supplementary figures and images for: The ‘Paths to everyday life’ (PEER) trial – a qualitative study of mechanisms of change from the perspectives of individuals with mental health difficulties participating in peer support groups led by volunteer peers
Source: BMC Psychiatry. 2024 Aug 13;24:555. doi: 10.1186/s12888-024-05992-w (PMC11321162; doi:10.1186/s12888-024-05992-w)

## Additional file 1: Initial program theory of the 'Paths to Everyday Life' (PEER) intervention

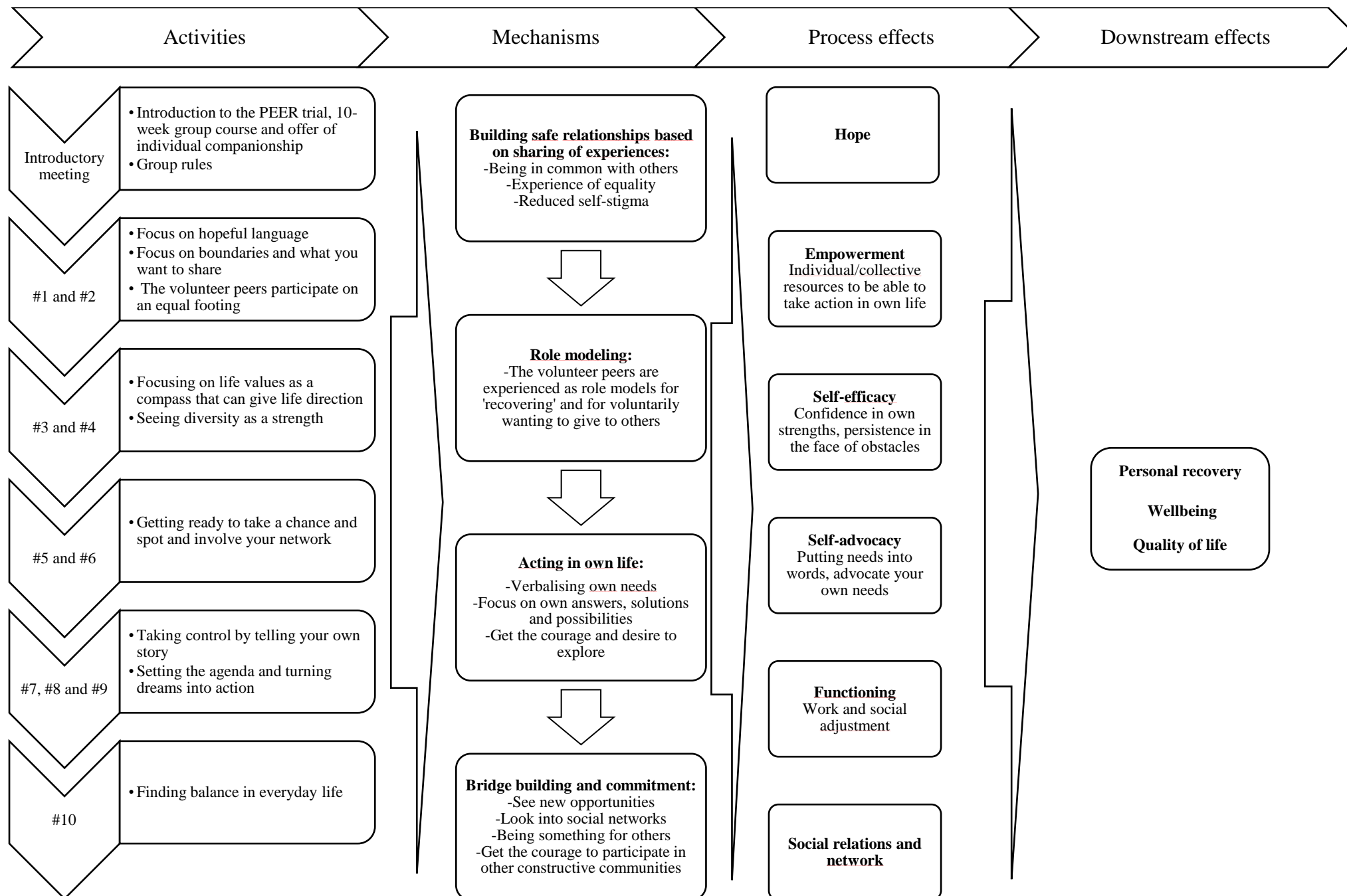

Supplement: Supplementary file 1 — Supplementary Material 1 [file 12888_2024_5992_MOESM1_ESM.pdf]
