# Supplementary material for: The ‘Paths to everyday life’ (PEER) trial – a qualitative study of mechanisms of change from the perspectives of individuals with mental health difficulties participating in peer support groups led by volunteer peers
Source: BMC Psychiatry. 2024 Aug 13;24:555. doi: 10.1186/s12888-024-05992-w (PMC11321162; doi:10.1186/s12888-024-05992-w)
Supplement: Supplementary file 2 — Supplementary Material 2 [file 12888_2024_5992_MOESM2_ESM.docx]

**Additional file 2:** Semi-structured interview guide for critical realist inspired interviews with participants in the group-based “Paths to Everyday life” (PEER) intervention

Before the start of the interview the interviewer presents her/himself and the informed consent is collected through an explanation of the interview and signing of the consent form. The interview is introduced by informing where the toilet is, how long the interview is going to last, how and why the questions are asked the way they are.

Articulate that the same group rules from the groups are applicable i.e., the participants should only share what they wish to share, and all participants has the right to withdraw from sharing. Questions can be rearticulated if necessary.

**Instructions for reading the interview guide:** Text in grey boxes, is only for the interviewer to connect and understand the questioning in relation to the program theory. Text in bullet points and in ***bold*** are direct questions to the participants. Text only in *italics* is introductory questions and in-depth sub-questions.

| **Semi-structured interview guide** | |
| --- | --- |
| **Purpose** | **Questions and/or explanations** |
| Setting the scene and introduction to program theory |  |
|  | Presentation of the interviewer and setting the scene for the interview situation (Information for participants, statement of consent, pauses etc.) |
|  | Turning on the Dictaphone – paying attention in placing it close to the participant. |
|  | Explanation of program theory - based on the printed group course program and outline (drawing by hand) the program theory in general terms. |
| Introduction |  |
|  | - ***Do you have any question or comments? – something you thought of when I talked about the program theory?*** |
|  | - ***Why were you interested in this group course?*** |
| Context |  |
| - Registration | - ***Why did you choose to sign up?*** |
| - Background | - ***We know very little about those who choose to participate in the group course. Can you tell a little about you background? Such as your age if you have children and where you live? Are there other things about you that are important to know, to better understand you and your current situation and why you chose to sign up to the group course?*** |
| - Education | - ***Can you tell a little about your education (primary school, higher education etc.) and potential work?*** |
| - Previous experiences | - ***We are curios if previous experience with participating in group courses means something to how it has been to participate in this group course. So if you are willing to share we would be interested in hearing a bit about whether you previously has participated in anything similar?*** - ***Have been in therapy either at a psychologist or in the mental health hospital?*** - ***Have you received any support from social services in the municipality? Do you think this had any importance for your choice to participate in the group course and how you experienced the group?*** |
| Mechanisms | Exploration if mechanisms are present and works as intended, as well as if some are missing or wrong in relation to creating the process outcomes. |
| 1. **Building safe relationship based on sharing experiences** | Building safe relationships is based on the theory that it can help create social relationships, hope and be a condition that must be present for the subsequent mechanisms to be activated. |
|  | *As I talked about in the theory of change, we believe that being together in the group with others who also have experiences with having some difficulties can help to create safe relationships and safety in the group. Therefore, we would like to explore whether that is the case.*   - ***I would like to hear if meeting others with experiences of having a difficult time, or experiences of trying to feel different, created an experience of safety for you in the group? How/how didn’t you experience it?*** - *For example, we are curious about the fact that the group sessions began by checking in, i.e., you shared a bit about how you had felt during the week - how did you experience it? What did you think about it?* - *Or maybe something completely different should have been present before you were safe. What could that have been?* |
| - Experience of being equal with others | *The idea behind this group, where to share experiences, and to give the opportunity to experience having something in common with others and that you are equal.*   - ***So how did you experience being in the group and having to share experiences?*** *What was difficult? What worked well?* |
| - Experience of equality | *We would like to explore more about peer support and whether it can create equality and how it happens in this intervention.*   - ***How did you experience the other group participants and the group as a whole? How were you similar? How were you different? What did that mean to you?*** - ***How did you experience the other group participants and the group facilitators in relation to sharing with each other?*** |
| 1. **Role modelling** | Peers as a role model is based on the theory that peers can be a source of hope and create a different sense of safety and connection than others you meet. That meeting other people who also have lived experience with mental illness and recovery can create an equal relationship, where it is easier to talk about what you need. |
| - Peers are experienced as role models for recovery and for voluntarily wanting to give | *We have a notion that the group leaders can act as role models both in relation to the possibility to get better/feel differently and to be able to do something like leading a group, but also that it can be valuable to voluntarily give something back to others.*   - ***Therefore, I am curious about how you experienced the two group leaders in relation to this?*** - ***We are curious about the fact that the group leaders were volunteers - was that something that meant anything to you?*** - ***What can volunteering do – for you?*** |
| 1. **Acting in own life** | Acting in own life is based on the theory that creating a focus on speaking one's own case, trying new things, finding out what is important to oneself can help to promote taking acting in own life and new views on own resources and abilities. |
| - Focus on own answers, solutions, and opportunities - Express own needs - Act in your own life | *We have a notion that noticing that you have things you can do, as well as that you have things you find important can help you to clarify your needs and help you to find the right answers and solutions for yourself. This can help you gain greater confidence in your own abilities, and that you can speak up for yourself and gain more power and action in your own life.*   - ***We are therefore curious about whether some of the group sessions and being in a group have contributed to changes for you? Or if you experienced something completely different in the group?*** - ***Can you tell me a little bit about whether the group has helped you to clarify what is important to you and what your needs are? Has it, for example, become clearer to you what you want?*** - ***Have you found the courage to do some of the things you want to do?***   • If not – why do you think that could be? What have you been missing?  • If so – what options do you have to act on it? And what steps were needed? |
| 1. **Bridging and engagement** | Building bridges and engagement in your own life and in offers you receive are based on the theory that trying new things, seeing your own resources, creating and involving your network - can lead to increases in the ability to function socially, expand network and generally increase the quality of life. |
| - New options for action | *We are interested in exploring the intervention element about trying new things and focusing on how to use your network and possibly mutually be something for your network in combination with the fact that you have become clear about what needs you have, can help create a bridge to new opportunities and actions.*   - ***I am interested in your experiences and perceptions and whether you have experienced new opportunities or things you would like to do?*** |
| - Social network - Be something for others | - ***Another thing we would like to explore more is whether being in this peer support group has created changes in your network and social relationships? It can be both an awareness of how your network can support you or how you can support others?*** - ***What does that change consist of and if so, how, and what helped to create a change?*** |
| - Exploration of the participant's perspective on the most important thing in the sessions | - ***I have asked about various things. However, we would like to learn from you what you think is the most important thing you will bring with you from this group course? It can both be negative or positive?*** |
| **Outcome process and impact** |  |
| **Unintended/unexpected effects** | *We do not believe that we have been able to create a comprehensive theory about what can happen during such a process here and therefore we would very much like to explore whether there has been something else, which we have overlooked.*   - ***Has the group course created any changes - good or bad - that you have not yet talked about and that you think is important to teach me about?*** |
| **Closing** | - ***We are about to finish but it is important to us if there is anything else that you consider important to tell us?*** |
|  |  |
| **End of interview** | |

Turn off Dictaphone. Say thank you for participating. Remind them that they can contact us by phone or email if they think of anything or there is something they regret having said.
